# Integrative Analysis of Terpenoid Profiles and Hormones from Fruits of Red-Flesh Citrus Mutants and Their Wild Types

**DOI:** 10.3390/molecules24193456

**Published:** 2019-09-23

**Authors:** Cuihua Liu, Min He, Zhuang Wang, Juan Xu

**Affiliations:** 1 College of Horticulture, Northwest A&F University, Yangling 712100, China; liuch@nwafu.edu.cn; 2 Key Laboratory of Horticultural Plant Biology (Ministry of Education), College of Horticulture and Forestry, Huazhong Agricultural University, Wuhan 430070, China

**Keywords:** carotenoids, aromas, bitter compounds, hormones, citrus

## Abstract

In citrus color mutants, the levels of carotenoid constituents and other secondary metabolites are different in their corresponding wild types. Terpenoids are closely related to coloration, bitterness, and flavor. In this study, terpenoid profiles and hormones in citrus fruits of two red-flesh mutants—Red Anliu orange and Red-flesh Guanxi pummelo—and their corresponding wild types were investigated using GC/MS, HPLC, and LC-MS/MS. Results showed that Red Anliu orange (high in carotenoids) and Anliu orange (low in carotenoids) accumulated low levels of limonoid aglycones but high levels of monoterpenoids; conversely, Red-flesh Guanxi pummelo (high in carotenoids) and Guanxi pummelo (deficient in carotenoids) accumulated high levels of limonoid aglycones but low levels of monoterpenoids. However, isopentenyl diphosphate was present at similar levels. A correlation analysis indicated that jasmonic and salicylic acids might play important roles in regulating terpenoid biosynthesis. Additionally, the similarities of carotenoid and volatile profiles between each mutant and its corresponding wild type were greater than those between the two mutants or the two wild types. The flux balance of terpenoid metabolism in citrus fruit tends toward stability among various citrus genera that have different terpenoid profiles. Bud mutations could influence metabolite profiles of citrus fruit to a limited extent.

## 1. Introduction

In citrus, the ability to accumulate anthocyanins is not a universal feature [1]; blood orange (*Citrus sinensis*) and purple pummelo (*Citrus maxima*) are two rare citrus accessions characterized by high anthocyanin accumulation in mature fruits [1]. Usually, the main pigments of citrus fruit are carotenoids [1,2]. Fruit color is an important indicator of exterior and inner quality levels, including nutrition and flavor [3]. Usually, the juice sacs of citrus fruit are yellow or orange; however, an increase in color mutants of citrus has been reported—for example, Shara orange [4], Cara Cara navel orange [5], Red Anliu orange (R-An) [6], Chuhong pummelo [7], and Red-flesh Guanxi pummelo (R-GX) [8]. Carotenoid and primary and secondary metabolite (e.g., sugars, acids, and flavonoids) constituents in color mutants are different from those of wild types [6,9].

In addition to tetraterpenoid carotenoids, monoterpenoid and sesquiterpenoid volatiles, and triterpenoid bitter compounds are important secondary metabolites of citrus fruit [7]. These terpenoids play key roles in fruit qualities, pollinator attraction, plant defense, and interactions with the surrounding environment [10]. Phytohormones of abscisic acid (ABA) and strigolactones are also synthesized by the terpenoid pathway [11,12]. ABA is one of the important hormones that regulate various aspects of fruit ripening. For example, respiratory metabolism, pigment biosynthesis, color changes, phenolic metabolism, nutritional contents, cell wall metabolism, fruit softening, and sugar and acid metabolism [13,14]. Recently, crosstalk among hormones have been studied [15,16]. Studies show that jasmonic acid (JA) and salicylic acid (SA) affect the maturation or ripening and related phenotypes of non-climacteric fruit and climacteric fruit [17,18,19,20].

Therefore, it is necessary to study the profiles and relationships of terpenoids and phytohormones in fruits of red-flesh citrus mutants. For this purpose, two red-flesh citrus mutants—R-An orange and R-GX pummelo—and their corresponding wild types were used in this study and their terpenoid profiles and constituents of phytohormones were investigated. Subsequently, the equivalent quantities of substrates for terpenoids were calculated and the effects of hormones and red-flesh bud mutations on terpenoid profiles were also revealed.

## 2. Results

In this study, two red-flesh citrus varieties and their respective wild types were used to explore the characteristics of phytohormones and terpenoid metabolites, including carotenoids, volatiles, and limonoid aglycones.

### 2.1. Carotenoids

In total, 11 carotenoids—violaxanthin, 9-*Z*-violaxanthin, lutein, α-carotene, β-carotene, phytonen, antheraxanthin, zeaxanthin, β-crytoxanthin, lycopene, and γ-carotene—were detected in juice sacs (Table 1).

Between the color mutations and their corresponding wild types, the most distinctive differences were in the accumulated levels of lycopene and total carotenoids in the juice sacs. Lycopene in R-An and R-GX reached levels of 130.52 and 591.21 µg/g, respectively, which accounted for 84.44% and 94.97% of total carotenoids, respectively. However, lycopene was undetected in An and only a trace level was detected in GX. In addition, the β-carotene level was significantly higher in red-flesh mutants than that in wild types. R-An and R-GX accumulated 2.21 µg/g and 22.21 µg/g of β-carotene, respectively, which represented 0.13 µg/g and a trace level in An and GX, respectively. Additionally, the 30.56 µg/g of γ-carotene accumulated in R-GX was the second highest carotenoid level, while it was undetected in the juice sacs of the other three samples.

The levels of total carotenoids in wild types were considerably lower than in red-flesh citrus varieties. The quantity of total carotenoids in R-An was 154.58 µg/g, which was 4.37-fold greater than in An. For R-GX, the total carotenoid content was 622.55, which was 202.13-fold greater than in GX.

### 2.2. Limonoid Aglycones

Among juice sacs, the concentrations of limonin, nomilin, and total Limonoid aglycones (LAs) in R-An were significantly higher than those in An. However, limonin, nomilin, and total LA levels in R-GX were lower than those in GX, but only the nomilin contents were significantly different (Table 2).

Importantly, the amounts of limonin, nomilin and total LAs in R-GX and GX were far greater than those in R-An and An. The concentrations of limonin in R-GX and GX were 1,439.15 and 1,722.79 µg/g, respectively, while those in R-An and An were 561.89 and 449.16 µg/g, respectively. Similarly, the concentrations of nomlin in R-GX and GX were 327.78 and 426.01 µg/g, respectively, while those in R-An and An were 13.20 and 7.02 µg/g, respectively. Compared with 575.09 and 456.18 µg/g of total LAs in R-An and An, respectively, 1,766.93 and 2,148.80 µg/g of total LAs were detected in R-GX and GX, respectively. On the basis of the concentrations of LAs in juice sacs, R-An and An had low-LA-containing fruit, while R-GX and GX had high-LA-containing fruit.

### 2.3. Volatiles

In the study, 71 volatiles were detected, among which 45 volatiles were positively identified with the assistance of authentic standards and 26 were tentatively identified. These volatile compounds were classified into 12 groups: 17 monoterpenes, nine monoterpene alcohols, four monoterpene aldehydes, two monoterpene esters, four monoterpene oxides, 17 sesquiterpenes, three sesquiterpene alcohols, two sesquiterpene aldehydes, one sesquiterpene ketone, one sesquiterpene oxide, four alcohols, and seven aldehydes (Table 3). 

The numbers of volatiles detected in R-An, An, R-GX, and GX were 66, 65, 33, and 31, respectively. The number of volatiles with significant differences between R-An and An was 33, among which 20 volatiles had significantly higher levels in R-An than in An. Only the contents of 10 volatiles in R-GX were significantly different from in GX, among which the contents of seven volatiles were significantly higher in R-GX than in GX. Germacrene D was the unique volatile that was significantly present in greater amounts in R-An and R-GX than in their wild types. However, the contents of monoterpenes, monoterpene aldehydes, monoterpene oxides, monoterpene esters, monoterpenoids, and total volatiles in both red-flesh mutants, R-An, and R-GX, were greater than in their wild types (Table S2).

Additionally, the total volatile contents of R-An and An were 8,403.64 and 7,118.58 µg/g, which were far greater than in R-GX (1181.91 µg/g) and GX (939.22 µg/g), which was mainly caused by a sharp decrease in d-limonene contents in R-GX and GX. The contents of d-limonene in R-An and An were 7846.10 and 6647.90 µg/g, respectively, while in R-GX and GX the contents were 878.30 and 656.70 µg/g, respectively (Table 3). Correspondingly, the percentages of d-limonene in R-GX and GX were 74.31% and 69.92%, respectively, while the values were 93.37% and 93.39% in R-An and An, respectively. However, the percentages of total monoterpenes in four citrus varieties were very similar, ranging from 96.54% in GX to 97.50% in An, which was caused by increases in the β-myrcene contents in R-GX and GX. The contents of β-myrcene in R-GX and GX were 243.72 and 226.04 µg/g, respectively, but the β-myrcene contents were 158.44 and 15.91 µg/g in R-An and An, respectively. As a result, the quantities of d-limonene and β-myrcene increased to 93.99–95.25% in the four citrus varieties. Additionally, monoterpene alcohols, monoterpene aldehydes, sesquiterpenes, and monoterpenes were also the main volatiles in citrus fruit (Figure S1).

Thus, the percentages of monoterpenes in the citrus fruit used in the study were similar, while the absolute amounts of total monoterpenes were very different. According to the total monoterpene contents, R-An and An had high-level monoterpene-containing fruit, while R-GX and GX were low-level monoterpene-containing fruit.

### 2.4. Isopentenyl Diphosphate (IPP) Content Analysis

**Figure 1:**
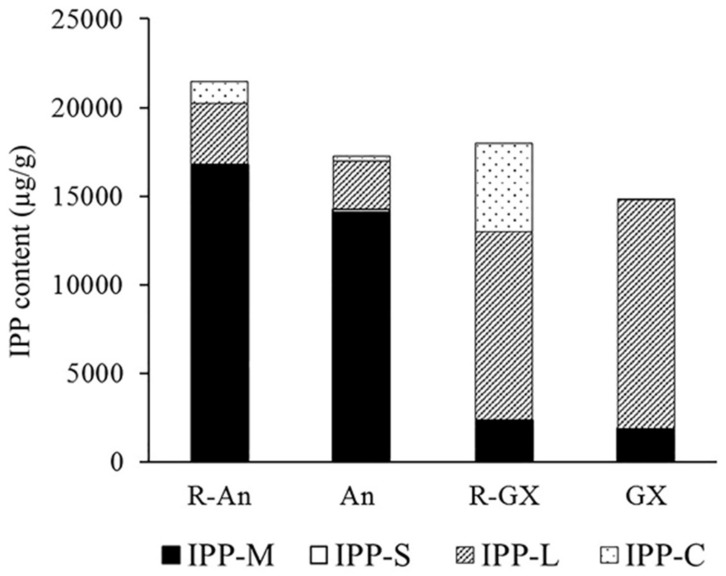
Total quantities of isopentenyl diphosphates (IPPs) used as substrates by four groups of terpenoids (µg/g). IPP-M, the quantities of IPPs converted from total monoterpenoids multiplied by 2; IPP-S, the quantities of IPPs converted from total sesquiterpenoids multiplied by 3; IPP-L, the quantities of IPPs converted from total limonoid aglycones multiplied by 6; IPP-C, the quantities of IPPs converted from total carotenoids multiplied by 8. R-An, Red Anliu orange; An, Anliu orange; R-GX, Red-flesh Guanxi pummelo; GX, Guanxi pummelo.

In this study, odor-related monoterpenoid and sesquiterpenoid volatiles, bitter limonoid aglycones of triterpenoids, and color-related carotenoids of tetraterpenoids were identified. IPP and dimethylallyl diphosphate, as their common precursors, could be interconverted with the assistance of isopentenyl diphosphate delta-isomerase in plastids. Therefore, the IPP contents were calculated based on quantities of various terpenoids. As shown in Figure 1, the total IPP contents ranged from 14,811.39 µg/g in GX to 21,474.96 µg/g in R-An, which were similar. However, the total IPPs of R-An and An were dominated by IPP converted from monoterpenoids, whereas the total IPPs of R-GX and GX were dominated by IPP converted from LAs. A partial correlation analysis showed that the correlation coefficient between total monoterpenoids and total LAs was −0.97 (*p* < 0.05). This indicated that a general balance and a stable metabolic flux in the terpenoid biosynthetic pathway were maintained in citrus fruit.

### 2.5. Phytohormones in Citrus Fruits

In the juice sacs of citrus fruit used in this study, four phytohormones were detected, ABA, IAA, JA, and SA. Interestingly, the ABA contents in both red-flesh mutants were significantly lower than those in their corresponding wild types, respectively (Table 4). ABA dominated the phytohormone profiles of all the juice sac samples used in the studies, with the ABA contents ranging from 97.40% in R-GX to 97.82% in GX.

IAA was only detected in An at 17.35 ± 0.51 ng/kg. No significant difference was detected in the JA contents between R-An and An, while it was significantly higher in R-GX than in GX. The SA concentrations in the juice sacs ranged from 12.24 ± 2.85 ng/kg in An to 19.38 ± 6.82 ng/kg in GX; no significant differences were detected between either R-An and An or R-GX and GX (Table 4).

### 2.6. The Correlation Between Plant Hormones and Terpenoids

**Figure 2:**
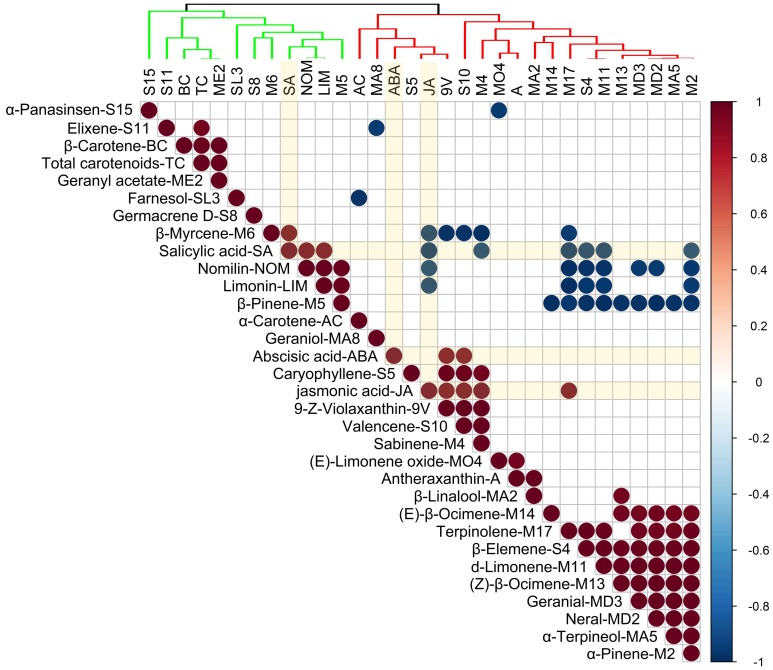
Correlation analysis using the main compounds in this study.

The correlation matrix was calculated based on the levels of 29 terpenoids and three hormones that appeared in all the samples used in the study. As shown in Figure 2, ABA was positively correlated with 9-*Z*-violaxanthin and valencene. JA was negatively correlated with β-myrcene, limonin, and nomilin, and positively correlated with 9-*Z*-violaxanthin, valencene, sabinene, and terpinolene. SA was positively correlated with β-myrcene, limonin and nomilin, and negatively correlated with sabinene, terpinolene, β-elemene, α-pinene, and d-limonene. This indicated that JA and SA might be important hormones for regulating terpenoid metabolism in citrus fruit.

### 2.7. Principal Component Analysis Based on Carotenoids and Volatiles

**Figure 3:**
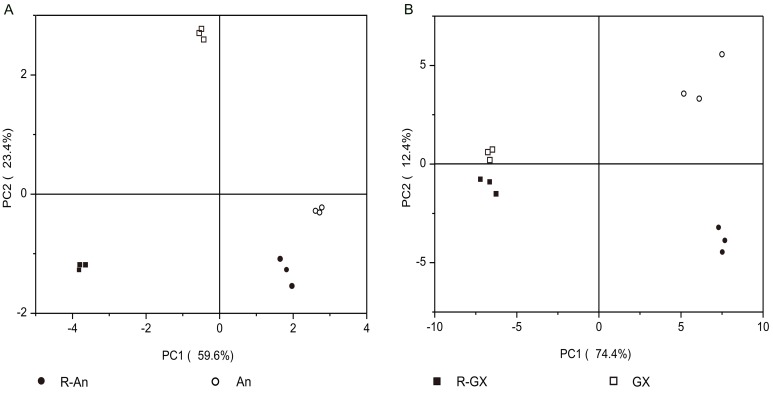
Principal component analysis (PCA) score plot. (**A**) PCA score plot of carotenoids; (**B**) PCA score plot of volatile compounds. R-An, Red Anliu orange; An, Anliu orange; R-GX, Red-flesh Guanxi pummelo; GX, Guanxi pummelo

To characterize the red-flesh mutants and their corresponding wild types, a principal component analysis (PCA) was employed using the carotenoids (Figure 3A) and volatile compounds (Figure 3B). As shown in Figure 3, the first two components in Figure 3A,B explained up to 83.0% and 86.8% of the variance, respectively. The four citrus fruits were completely separated in the PCA plots. Importantly, R-An and An, and R-GX and GX, could be separated on the PC1 axis, explaining 59.6% and 74.4% of the variance in Figure 3A,B, respectively. Additionally, the heatmap dendrogram analysis also suggested that R-An and An clustered together, as did R-GX and GX (Figure S2). Furthermore, both red-mutants (R-An and R-GX) and both wild types (An and GX) were separated clearly on the PC2 axis, explaining 23.4% and 12.4% of the variance in Figure 3A,B, respectively. Their loading plots are shown in Figure S3.

## 3. Discussion

### 3.1. Equivalent Quantities of IPP in the Terpenoid Biosynthetic Pathway Are Prone to Stability

Two different citrus species of fruits, sweet oranges of R-An and An (*Citrus sinensis*), together with pummelos of R-GX and GX (*Citrus maxima*), were employed in the study and their terpenoid profiles were very different. Both R-An (with a high carotenoid content) and An (with a low carotenoid content) accumulated low levels of LAs but high levels of monoterpenoids; conversely, R-GX (with a high carotenoid content) and GX (which was deficient in carotenoids) accumulated high levels of LAs but low levels of monoterpenoids. However, their levels of equivalent quantities of IPP, the common substrate of terpenoids, were similar; that is, the roughly similar amounts of isoprene carbon were produced by them. This suggested that the flux balance of terpenoid metabolism in citrus fruit was prone to stability among various citrus genera that had different terpenoid profiles, and the variation of terpenoid profiles among these citrus genera was caused by the partitioning of carbon in downstream isoprenoid pathways but not increased flux through the isoprenoid pathway, which agreed with previous studies. Liu et al. [7] found a total capacity or a balance in the production of various terpenoids in pummelo. Li et al. [22] revealed that the plastid methylerythritol phosphate pathway was enhanced in Niurouhong tangerine (*Citrus reticulata* Blanco), which has a beef-red color, resulting from a great accumulation of β-cryptoxanthin and β-carotene, and compared with its wild type, the cytosolic mevalonic acid pathway was suppressed in Niurouhong tangerine. Recent developments are predominantly driven by experimental and computational advancements, producing a network-wide metabolic flux and metabolome maps, which enable the determination of cellular metabolism and the estimation of metabolic flux in vivo by applying a natural isotope correction of the MS/MS measurements [23]. Wada et al. [24] constructed a mevalonic acid (MVA)-producing strain of *Escherichia coli* by introducing acetoacetyl-CoA synthase/3-hydroxy-3-methylglutaryl-CoA (HMG-CoA) reductase and HMG-CoA synthase genes from *Enterococcus faecalis*. The ^13^C-metabolic flux analysis revealed that the MVA yield of the engineered strain was close to the upper limit, but the flux levels in acetate formation and the TCA cycle in the engineered strain were lower than those in the control strain. This indicated that the metabolic flux levels in these organisms are prone to stability.

### 3.2. Effects of Hormones on Terpenoid Metabolism

Among the four hormones investigated in the study, only the ABA contents of both red-flesh mutants were significantly lower than those in wild types, which agreed with the results in Niurouhong tangerine (*Citrus reticulata* Blanco) and Pinalate (*Citrus sinensis* L. Osbeck), two previously reported citrus color mutants [22,25]. A significant decrease in ABA—a downstream metabolite of the carotenoid biosynthetic pathway—in mutants that had high levels of some carotenoids was very common, and the mechanism should be further studied.

JA and SA were significantly correlated with a variety of terpenoids, which indicated that JA and SA might be important hormones involved in regulating terpenoid biosynthesis in citrus fruit. In *Panax ginseng* adventitious roots treated with SA, the contents of JA and some terpenoids—such as farensol, isochiapin B sesquiterpenoids, champhor, and cineole monoterpenoids—are higher [26]. SA and terpenoid volatiles, including (*E*)-β-ocimene, linalool, methyl salicylate, indole, caryophyllene, β-elemene, and α-farnesene, are accumulated in tobacco plants infected with the avirulent strain *Pseudomonas syringae* pv. *Maculicola* ES4326 or pv. *Tomato* DC3000 [27]. The biosynthesis of (*E*)-β-ocimene, linalool, β-caryophyllene, and germacrene D can be induced by methyl jasmonate in grape leaf [28]. Similar results were also reported in sweet basil, tomato, and *Catharanthus roseus* [29,30,31,32]. The biosynthetic and regulatory mechanisms of terpenoids in citrus should be further studied.

### 3.3. Effects of Red-Flesh Bud Mutations on Metabolite Profiles

Both the R-An and R-GX red-flesh mutants used in the study were spontaneous bud mutations [6,8]. Compared with wild types, multiple metabolite profiles of red-flesh mutants varied, including carotenoids, volatiles, bitter compounds, hormones, flavonoids, sugars, and acids in this study and previous studies [6,7,8,9,22,25]. Additionally, the similarities in carotenoid and volatile profiles between each mutant and its corresponding wild type were greater than those between both mutants or both wild types (Figure 3 and Figure S2), which suggested that bud mutations could influence metabolite profiles of citrus fruit to some extent, but the influence was limited. Notably, R-An and R-GX highly accumulate lycopene in their fruit pulp, which demonstrate distinctive red-flesh and have high economic and health value. Therefore, these red-flesh mutants are more popular than their wild types by planters and consumers. Especially, R-GX accumulated considerably high contents of carotenoids and LAs, which have been reported to be responsible for a chemopreventive and therapeutic role in human health, as demonstrated in large cohort and case control studies of cancer, heart disease, and many other diseases [33]. It is expected that R-An and R-GX will be used more widely than their wild types for table fruits or red-fleshed citrus juices as potential natural resources because of their higher nutritional and medicinal properties [34].

## 4. Materials and Methods

### 4.1. Plant Materials

Mature fruits of R-An and Anliu (An) orange (*Citrus sinensis*) and R-GX and Guanxi pummelo (GX) (*Citrus maxima*) were harvested in the winter of 2011. Detailed fruit sample information is listed in Table 5.

The sample preparations for flavedo and juice sacs were conducted in accordance with Liu et al. [7,21]. The flavedo around the equatorial plane of fruits was snap-frozen in liquid nitrogen without touching the inner part of the fruit. A portion of the flavedo was stored at −80 °C for further volatile compound extractions and analysis. The juice sacs were lyophilized (Heto Lyolab 3000, Heto-Holten A/S, Allerød, Denmark) and stored at −80 °C for carotenoid, bitter compound, and hormone extractions. Three independent biological replicates were prepared for each sample.

### 4.2. Standards and Reagents

For the carotenoid analysis, antheraxanthin, α-carotene, β-cryptoxanthin, all-trans-lutein, phytoene, and all-trans-violaxanthin were obtained commercially from CaroteNature (Lupsingen, Switzerland); β-carotene and all-trans-lycopene were obtained from Sigma Co. Ltd. (St Louis, MO, USA).

For the bitter compound analysis, limonin, nomilin, and naringin of high-performance liquid chromatography (HPLC) grade were purchased from Sigma Co. Ltd.

For the plant hormone analysis, indol-3-acetic-2,2-[^2^H_5_] acid ([^2^H_5_]IAA), indole-3-actic acid (IAA), 2-hydroxy-benzoic acid-[^2^H_4_] ([^2^H_4_]SA), SA, 3-oxo-2-(cis-2-pentenyl) cyclopentane-1-[^2^H_2_] acetic acid ([^2^H_2_]JA), JA, 2-*cis*, 4-trans-abscisic acid-[^2^H_6_] ([^2^H_6_] ABA), and ABA were purchased from OlChemIm Ltd. (Olomouc, Czech Republic).

For the volatile analysis, the internal standard of methyl nonanoate was obtained from Sigma Co. Ltd. A standard series of C_7_–C_30_ saturated alkanes from Supelco (Bellefonte, PA., USA) were used for retention index (RI) determination. The sources of the volatile standards are listed in Table 1 of a previously published paper (Table S1) [21].

In addition, the following were used in the metabolite extraction solution: methyl tertbutyl ether (MTBE), methol, and acetonitrile (HPLC grade) from Tedia (Fairfield, CT, USA); and triethylamine (HPLC grade), ethanol, hexane, acetone, iso-propyl alcohol, dichloromethane, trichloromethane, and methanoic acid from Sinopharm Chemical Reagent Co., Ltd. (Shanghai, China).

### 4.3. Carotenoid Extraction with MTBE and HPLC Analysis

Carotenoids in fruit juice sacs and leaves were extracted in accordance with Liu et al. [6]. In total, 1 g of lyophilized sample was homogenized with 15 mL of extraction solution (hexane/acetone/ethanol = 2:1:1, *v*/*v*/*v*, containing 0.1 g/L butylated hydroxytoluene). After 12 h of saponification in darkness with 2 mL of KOH/water/methanol (10:25:75, *w*/*v*/*v*), the sample was rinsed with saturated NaCl solution until it became neutral. After evaporation under a vacuum, the residue was redissolved in 0.6 mL MTBE. Carotenoid extracts were separated by HPLC (Waters 1525, Waters Co., Milford, MA, USA) equipped with a YMC C30 carotenoid column (150 mm × 4.6 mm, 3 µm; YMC, Wilmington, NC, USA) and a 2996 photodiode array detector. Methanol:acetonitrile (1:3, *v*/*v*) and MTBE were employed as eluent A and B, respectively.

The following linear gradient program was used: 0–10 min, 95:5 A:B; 10–19 min, 86:14 A:B; 19–29 min, 75:25 A:B; 29–54 min, 50:50 A:B; 54–66 min, 26:74 A:B; 67 min, 95:5 A:B.

### 4.4. Limonoid Aglycone Extraction and HPLC Analysis

In accordance with Li et al. [35] and Liu et al. [7], without any modification, bitter compounds were extracted. Briefly, with the assistance of a FexIKA vario control (IKA-Werke GmbH and Co. KG, Staufen, German), lyophilized powder of 3 g of juice sacs or flavedo was extracted with 50 mL of dichloromethane. The extraction solution was collected and dried under vacuum in an Eppendorf 5301 concentrator (Eppendorf, Hamburg, Germany) after 15 cycles of Soxhlet extraction. Finally, the residue was resolved in 1 mL of acetonitrile. The HPLC equipment was the same as for the carotenoid analysis, except that a C18 HPLC column (150 mm × 4.6 mm, 5 μm; Agilent, Wilmington, DE, USA) was used. A 20 μL sample was injected into the HPLC and eluted with acetonitrile/100 mL L^−1^ methanol (40:60, *v*/*v*) at a flow rate of 1 mL min^−1^.

### 4.5. Plant Hormone Extraction and Analysis with LC-MS

Plant hormones were extracted in accordance with Pan et al. [36] and Ma et al. [37]. A 10 ng aliquot of [^2^H_5_]IAA, [^2^H_4_]SA, [^2^H_2_]JA, and [^2^H_6_] ABA were used as internal standards. Hormones were separated by HPLC (Agilent 1100, Agilent Technologies, Palo Alto, CA, USA) and measured using an HPLC-electrometer (Applied Biosystems, Foster City, CA, USA). The MS/MS conditions for each analyte was set in accordance with Pan et al. [36].

### 4.6. Volatile Compound Extraction and GC-MS Analysis

In citrus fruit, volatile compounds are mainly accumulated in the oil glands of the peel [38]. Thus, citrus peel was used for volatile compound extractions. In accordance with Liu et al. [21], 3 g of peel powder was extracted in 15 mL MTBE containing 400 μg of methyl nonanoate as an internal standard. The extraction was carried out in an ultrasonic cleaner FS60 (Fisher Scientific, Pittsburgh, PA, USA) for 1 h and the supernatant was collected and concentrated to 1.4 mL under a gentle N_2_ stream.

An aliquot of 1 μL was analyzed by TRACE GC Ultra GC coupled with an ISQ mass spectrometer (Thermo Fisher Scientific, Waltham, MA, USA) and equipped with a TRACE^TM^ TR-5 MS column (30 m × 0.25 mm × 0.25 μm, Thermo Scientific, Bellefonte, PA, USA). The temperatures of the injection port, ion source, and MS transfer line were maintained at 250, 260, and 280 °C, respectively. Helium was used as a carrier gas with a split ratio of 50:1 at 1 mL/min. The column temperature program was initiated with 40 °C for 3 min, followed by a ramp of 3 °C/min until 160 °C for 1 min, then at a rate of 5 °C/min until 200 °C for 1 min, and finally, the temperature was raised to 240 °C at a rate of 8 °C/min and maintained for 3 min. The *m*/*z* range of the MS scan was from 45 to 400 in positive electron ionization mode at an ionization energy of 70 eV.

### 4.7. Data Analysis

The identification and quantification of carotenoids, bitter compounds, and volatiles were performed in accordance with Liu et al. [7,39], while the identification and quantification of plant hormones were performed in accordance with Pan et al. [36].

Significant differences in compounds between each color mutant and its corresponding control were analyzed using Student’s t-test (*p* < 0.05) with SAS software (SAS Institute, Cary, NC, USA).

After autoscaling pretreatment with values of carotenoids and volatiles was done as described by van den Berg et al. [40], the package of Ggbiplot in R Version 3.5.1 software (http://www.r-project.org, R Development Core Team, Vienna, Austria) was applied to the PCA of carotenoids and volatile compounds. The package of Corrplot in R was used for the correlation analysis based on Pearson’s correlation method.

## Figures and Tables

**Table 1:** Carotenoids in juice sacs of red-flesh citrus mutants and their corresponding wild types (µg/g DW ^a^).

Compounds	Code	R-An ^b^	An	R-GX	GX
Violaxanthin	V	4.92 ± 0.37	9.37 ± 1.16 *	/	/
9-*Z*-Violaxanthin	9V	8.97 ± 0.85	18.98 ± 2.20 *	0.95 ± 0.02	0.86 ± 0.01 *
Lutein	L	0.82 ± 0.07	0.09 ± 0.07 *	/	/
α-Carotene	AC	0.98 ± 0.02	0.92 ± 0.03	0.89 ± 0.02	0.48 ± 0.01 *
β-Carotene	BC	2.21 ± 0.19	0.13 ± 0.01 *	22.21 ± 0.19	Trace *
Phytoene	P	/	/	6.62 ± 0.04	/ *
Antheraxanthin	A	5.22 ± 0.39	1.45 ± 0.30 *	0.21 ± 0.01	0.42 ± 0.05 *
Zeaxanthin	Z	/	/	1.06 ± 0.01	0.81 ± 0.01 *
β-Crytoxanthin	CR	0.93 ± 0.02	4.39 ± 0.37 *	/	0.83 ± 0.01 *
Lycopene	LY	130.52 ± 6.80	/ *	591.21 ± 9.65	Trace *
γ-Carotene	CC	/	/	30.56 ± 0.98	/ *
Total carotenoids	TC	154.58 ± 7.82	35.34 ± 3.84 *	622.55 ± 9.13	3.08 ± 0.34 *

Note: ^a^ DW, dry weight. ^b^ R-An, Red Anliu orange; An, Anliu orange; R-GX, Red-flesh Guanxi pummelo; GX, Guanxi pummelo; each value is the mean ± SE; /, not detected; Trace, compounds detected at a trace level. *, significant differences between the red-flesh mutant and its corresponding wild type at *p* < 0.05.

**Table 2:** Limonoid aglycones (LAs) in juice sacs of red-flesh citrus mutants and their corresponding wild types (µg/g DW ^a^).

Compounds	Code	R-An ^b^	An	R-GX	GX
Limonin	LIM	561.89 ± 46.41	449.16 ± 8.76 *	1439.15 ± 153.45	1722.79 ± 200.16
Nomilin	NOM	13.20 ± 1.99	7.02 ± 0.29 *	327.78 ± 36.16	426.01 ± 17.01 *
Total LAs	TLA	575.09 ± 48.39	456.18 ± 9.03 *	1766.93 ± 186.29	2148.80 ± 189.74

Note: ^a^ DW, dry weight. ^b^ R-An, Red Anliu orange; An, Anliu orange; R-GX, Red-flesh Guanxi pummelo; GX, Guanxi pummelo; each value is the mean ± SE. *, significant gnificant differences between the red-flesh mutant and its corresponding wild type at *p* < 0.05.

**Table 3:** Volatiles in the flavedo of red-flesh citrus mutants and their corresponding wild types (µg/g FW ^a^).

Compounds ^b^	Code	RI ^c^	RIx ^d^	R-An ^e^	An	R-GX	GX
Monoterpenes	M						
α-Thujene^T10^	M1	931	929	0.52 ± 0.06	0.84 ± 0.09 *	/	/
α-Pinene	M2	938	937	52.34 ± 0.18	40.52 ± 1.64 *	4.14 ± 1.14	3.33 ± 0.74
Camphene	M3	956	951	0.29 ± 0.02	0.26 ± 0.07	/	/
Sabinene	M4	979	974	58.70 ± 7.11	101.00 ± 7.44 *	3.09 ± 0.52	3.12 ± 0.21
β-Pinene	M5	982	979	2.98 ± 0.33	4.56 ± 0.29 *	9.56 ± 1.24	11.69 ± 0.81
β-Myrcene	M6	994	991	158.44 ± 4.13	115.91 ± 5.68 *	243.72 ± 82.07	226.04 ± 58.49
Pseudolimonene^T11^	M7	1008	1004	0.31 ± 0.05	0.13 ± 0.12	/	/
α-Phellandrene	M8	1011	1005	13.10 ± 2.38	13.71 ± 1.40	0.14 ± 0.25	/
α-Terpinene	M9	1023	1017	0.37 ± 0.07	0.44 ± 0.04	/	/
Sylvestrene^T11^	M10	1027	1027	0.15 ± 0.13	0.24 ± 0.09	/	/
d-Limonene	M11	1038	1030	7846.10 ± 436.16	6647.90 ± 347.94 *	878.30 ± 282.27	656.70 ± 183.73
β-Phellandrene^T10^	M12	1040	1031	0.60 ± 0.25	1.48 ± 0.16 *	/	/
(*Z*)-β-Ocimene	M13	1044	1038	0.54 ± 0.04	0.43 ± 0.11	0.26 ± 0.01	0.24 ± 0.05
(*E*)-β-Ocimene	M14	1055	1049	10.88 ± 1.07	9.20 ± 1.52	7.22 ± 0.18	5.51 ± 0.80 *
4-Carene^T11^	M15	1060	1009	0.33 ± 0.07	/ *	/	/
γ-Terpiene	M16	1066	1060	0.52 ± 0.09	0.62 ± 0.01	/	/
Terpinolene	M17	1090	1088	3.50 ± 0.69	3.69 ± 0.33	0.23 ± 0.02	0.12 ± 0.11
Monoterpene Alcohols	MA						
(*Z*)-Sabinene hydrate	MA1	1079	1077	2.53 ± 0.25	3.28 ± 0.41	/	/
β-Linalool	MA2	1107	1099	107.93 ± 4.81	38.65 ± 3.79 *	2.67 ± 0.92	2.57 ± 0.15
(*E*)-p-Mentha-2,8-dienol^T12^	MA3	1133	1123	0.49 ± 0.05	0.24 ± 0.04 *	/	/
4-Terpineol	MA4	1190	1177	0.65 ± 0.02	0.67 ± 0.10	/	/
α-Terpineol	MA5	1206	1189	10.70 ± 0.65	7.65 ± 1.03 *	1.15 ± 0.43	1.24 ± 0.25
(*E*)-Piperitol^T1^	MA6	1220	1208	/	/	0.14 ± 0.12	0.12 ± 0.11
Citronellol	MA7	1236	1228	1.55 ± 0.13	1.66 ± 0.25	/	/
Geraniol	MA8	1261	1255	2.03 ± 0.26	2.47 ± 0.54	1.47 ± 0.77	2.01 ± 0.25
p-Mentha-1(7),8(10)-dien-9-ol^T1^	MA9	1303	/	1.68 ± 0.10	0.82 ± 0.15 *	/	/
Monoterpene alDehydes	MD						
Citronellal	MD1	1164	1153	7.85 ± 0.38	5.27 ± 0.25 *	/	/
Neral	MD2	1251	1240	16.22 ± 0.58	12.01 ± 0.81 *	2.28 ± 0.77	1.56 ± 0.54
Geranial	MD3	1281	1270	22.28 ± 0.70	16.47 ± 1.60 *	2.87 ± 1.02	2.22 ± 0.83
Perillal^T13^	MD4	1291	1272	6.44 ± 0.27	2.49 ± 0.20 *	/	/
Monoterpene Esters	ME						
Neryl acetate	ME1	1366	1364	0.46 ± 0.06	0.30 ± 0.07 *	0.26 ± 0.04	/ *
Geranyl acetate	ME2	1385	1382	0.32 ± 0.28	0.27 ± 0.14	0.57 ± 0.08	0.26 ± 0.06 *
Monoterpene Oxides	MO						
(*Z*)-Linalool oxide	MO1	1078	1074	/	/	0.30 ± 0.07	0.09 ± 0.02 *
(*E*)-Linalool oxide	MO2	1093	1086	/	/	0.16 ± 0.14	/
(*Z*)-Limonene oxide	MO3	1143	1134	0.46 ± 0.04	0.39 ± 0.07	/	/
(*E*)-Limonene oxide	MO4	1148	1138	3.24 ± 0.09	0.21 ± 0.05 *	0.29 ± 0.07	0.27 ± 0.09
Sesquiterpenes	S						
δ-Elemene ^T13^	S1	1338	1338	/	/	1.95 ± 0.58	1.64 ± 0.14
Copaene ^T13^	S2	1380	1376	4.18 ± 0.37	2.26 ± 0.13 *	/	/
β-Cubebene ^T3^	S3	1391	1389	3.13 ± 0.18	1.59 ± 0.07 *	/	/
β-Elemene ^T13^	S4	1393	1391	1.17 ± 0.10	1.14 ± 0.05	0.93 ± 0.29	0.93 ± 0.17
Caryophyllene	S5	1424	1419	2.88 ± 0.10	6.55 ± 0.09 *	1.72 ± 0.22	0.75 ± 0.28 *
β-Farnesene	S6	1457	1457	0.84 ± 0.10	5.05 ± 0.29 *	/	/
α-Caryophyllene	S7	1461	1454	0.67 ± 0.03	0.50 ± 0.02 *	/	/
Germacrene D ^T2^	S8	1487	1481	2.85 ± 0.18	1.64 ± 0.08 *	11.59 ± 1.98	7.11 ± 2.05 *
γ-Selinene ^T3^	S9	1489	1481	0.41 ± 0.05	0.79 ± 0.16 *	/	/
Valencene	S10	1497	1492	8.89 ± 0.11	16.19 ± 0.62 *	2.34 ± 0.34	2.37 ± 0.52
Elixene ^T13^	S11	1500	1471	1.38 ± 0.05	1.10 ± 0.40	1.70 ± 0.20	1.22 ± 0.20 *
α-Muurolene ^T3^	S12	1505	1499	0.62 ± 0.12	0.50 ± 0.09	/	/
α-Farnesene ^T4^	S13	1508	1508	1.11 ± 0.16	12.86 ± 1.42 *	/	/
δ-Cadinene ^T13^	S14	1524	1524	4.91 ± 0.40	2.43 ± 0.20 *	/	/
α-Panasinsen ^T13^	S15	1525	1527	0.13 ± 0.22	0.69 ± 0.15 *	0.50 ± 0.13	0.60 ± 0.18
β-Sesquiphellandrene ^T4^	S16	1530	1524	0.27 ± 0.06	0.46 ± 0.12	/	/
Germacrene B ^T13^	S17	1566	1557	/	/	0.45 ± 0.39	0.49 ± 0.16
Sesquiterpene aLcohols	SL						
(*E*)-Nerolidol	SL1	1567	1564	0.99 ± 0.12	0.73 ± 0.16	/	/
Germacrene D-4-ol ^T3^	SL2	1585	1574	0.43 ± 0.03	0.51 ± 0.11	/	/
Farnesol	SL3	1727	1713	1.02 ± 0.09	0.89 ± 0.23	0.73 ± 0.66	4.82 ± 0.40 *
Sesquiterpene alDehydes	SD						
β-Sinensal ^T3^	SD1	1705	1695	1.31 ± 0.16	1.04 ± 0.43	/	/
α-Sinensal ^T3^	SD2	1767	1752	1.39 ± 0.53	2.46 ± 0.71	/	/
Sesquiterpene Ketone	SK						
Nootkatone	SK1	1824	1808	0.69 ± 0.12	1.47 ± 0.56	/	/
Sesquiterpene oxide	SO						
Caryophyllene oxide	SO1	1591	1581	0.58 ± 0.11	1.86 ± 0.31 *	/	/
ALcohols	AL						
(*Z*)-3-Hexenol	AL1	868	856	2.35 ± 0.34	2.36 ± 0.20	0.70 ± 0.09	0.73 ± 0.12
(*E*)-2-Hexenol	AL2	878	862	/	/	/	0.49 ± 0.12 *
Hexanol	AL3	881	868	0.94 ± 0.31	1.04 ± 0.06	0.06 ± 0.10	0.35 ± 0.08 *
Octanol	AL4	1081	1071	3.65 ± 1.01	2.91 ± 0.64	/	/
AlDehydes	AD						
3-Hexenal ^T7^	AD1	811	810	0.56 ± 0.40	0.67 ± 0.12	/	/
Hexanal	AD2	812	800	2.06 ± 0.59	1.76 ± 0.31	0.10 ± 0.17	0.21 ± 0.19
(*E*)-2-Hexenal	AD3	870	854	1.09 ± 0.14	0.71 ± 0.15 *	/	/
Nonanal	AD4	1116	1104	2.62 ± 0.57	1.62 ± 0.22 *	/	/
Decanal	AD5	1216	1206	12.84 ± 3.15	8.99 ± 1.73	0.33 ± 0.12	0.42 ± 0.05
Undecanal	AD6	1318	1307	0.88 ± 0.12	0.42 ± 0.11 *	/	/
Dodecanal	AD7	1418	1409	2.34 ± 0.52	1.61 ± 0.15	/	/
Monoterpenes	TM			8149.68	6940.94	1146.67	906.74
Monoterpene Alcohols	TMA			127.57	55.45	5.43	5.93
Monoterpene alDehydes	TMD			52.78	36.23	5.14	3.78
Monoterpene Oxides	TMO			3.70	0.60	0.75	0.36
Monoterpene Esters	TME			0.78	0.57	0.83	0.26
Sesquiterpenes	TS			33.40	53.74	21.17	15.12
Sesquiterpene aLcohols	TSL			2.43	2.13	0.73	4.82
Sesquiterpene alDehydes	TSD			2.70	3.49	/	/
Sesquiterpene Ketone	TSK			0.69	1.47	/	/
Sesquiterpene Oxide	TSO			0.58	1.86	/	/
Alcohols	TA			6.94	6.31	0.76	1.57
AlDehydes	TAD			22.38	15.79	0.43	0.64
MonoTerpenoids	TMT			8334.51	7033.79	1158.82	917.08
SesquiTerpenoids	TST			39.80	62.70	21.90	19.93
Non-Terpenoids	TNT			29.33	22.10	1.19	2.21
Total Volatiles	TV			8403.64	7118.58	1181.91	939.22

Note: ^a^ FW, fresh weight. ^b^ Tn, quantified by total ion current mode, while unlabeled compounds were quantified by selective ion monitoring mode, according to Table 1 in Liu et al. [21] (Table S1). ^c^ RI, retention index on a TR-5 column in the study. ^d^ RIx, retention index on a semistandard nonpolar column. Values were obtained from the NIST 2014 library (http://nistmassspeclibrary.com/). ^e^ R-An, Red Anliu orange; An, Anliu orange; R-GX, Red-flesh Guanxi pummelo; GX, Guanxi pummelo; each value is the mean ± SE; /, not detected. *, significant differences between the red-flesh mutant and its corresponding wild type at *p* < 0.05.

**Table 4:** Phytohormones in juice sacs of red-flesh citrus mutants and their corresponding wild types (ng/g DW ^a^).

	R-An ^b^	An	R-GX	GX
ABA	1428.49 ± 103.87	2242.52 ± 126.82 *	1089.35 ± 50.03	1276.14 ± 53.30 *
IAA	/	17.35 ± 0.51 *	/	/
JA	19.01 ± 4.32	23.02 ± 7.43	9.90 ± 0.38	9.04 ± 0.24 *
SA	13.28 ± 2.09	12.24 ± 2.85	19.12 ± 3.01	19.38 ± 6.82

Note: ^a^ DW, dry weight. ^b^ R-An, Red Anliu orange; An, Anliu orange; R-GX, Red-flesh Guanxi pummelo; GX, Guanxi pummelo; each value is the mean ± SE; /, not detected. *, significant differences between the red-flesh mutant and its corresponding wild type at *p* < 0.05.

**Table 5:** Sampling information for fruits of red-flesh citrus mutants and their corresponding wild types.

Cultivar	Flesh Color	Code	Production Area	Remarks
Red Anliu orange	Red flesh	R-An	Citrus Research Institute of Guangxi, Guilin, Guangxi province	Red-flesh mutant of Anliu orange
Anliu orange	Yellow flesh	An		Common sweet orange
Red-flesh Guanxi pummelo	Red flesh	R-GX	Fujian Academy of Agricultural Sciences, Xiamen, Fujian province	Red-flesh mutant of Guanxi pummelo
Guanxi pummelo	Pale yellow flesh	GX		Common white-flesh pummelo

## Supplementary Materials

The following are available online, Figure S1: Classes and proportions of volatile compounds in fruit flavedo of Red Anliu orange (A), Anliu orange (B), Red-flesh Guanxi pummelo (C), and Guanxi pummelo (D). M: monoterpenes; MA: monoterpene alcohols; MD: monoterpene aldehydes; ME: monoterpene esters; MO: monoterpene oxides; S: sesquiterpenes; SL: sesquiterpene alcohols; SD: sesquiterpene aldehydes; SK: sesquiterpene ketone; SO: sesquiterpene oxide; AL: alcohols; AD: aldehydes. Figure S2: Heatmap dendrogram analysis based on 32 common compounds detected in fruit of four citrus varieties. S15: α-panasinsen; S11: elixene; BC: β-carotene; TC: total carotenoids; ME2: geranyl acetate; SL3: farnesol; S8: germacrene D; M6: β-myrcene; SA: salicylic acid; NOM: nomilin; LIM: limonin; M5: β-pinene; MA8: geraniol; ABA: abscisic acid; S5: caryophyllene; 9V: 9-*Z*-violaxanthin; S10: valencene; M4: sabinene; AC: α-carotene; MO4: (*E*)-limonene oxide; A: antheraxanthin; MA2: β-linalool; JA: jasmonic acid; M17: terpinolene; M14: (*E*)-β-ocimene; S4: β-elemene; M11: d-limonene; M13: (*Z*)-β-ocimene; MD3: geranial; MD2: neral; MA5: α-terpineol; M2: α-pinene. Figure S3: Principal component analysis (PCA) loading plot. A, PCA loading plot of carotenoids; B, PCA loading plot of volatile compounds. R-An, Red Anliu orange; An, Anliu orange; R-GX, Red-flesh Guanxi pummelo; GX, Guanxi pummelo. Table S1: Authentic volatile standards used and their equations of standard curves in GC-MS analysis. Table S2: Ratios of volatiles in the flavedo between red-flesh citrus mutants and their corresponding wild types.
