# Supplementary figures and images for: Integrative Analysis of Terpenoid Profiles and Hormones from Fruits of Red-Flesh Citrus Mutants and Their Wild Types
Source: Molecules. 2019 Sep 23;24(19):3456. doi: 10.3390/molecules24193456 (PMC6804237; doi:10.3390/molecules24193456)

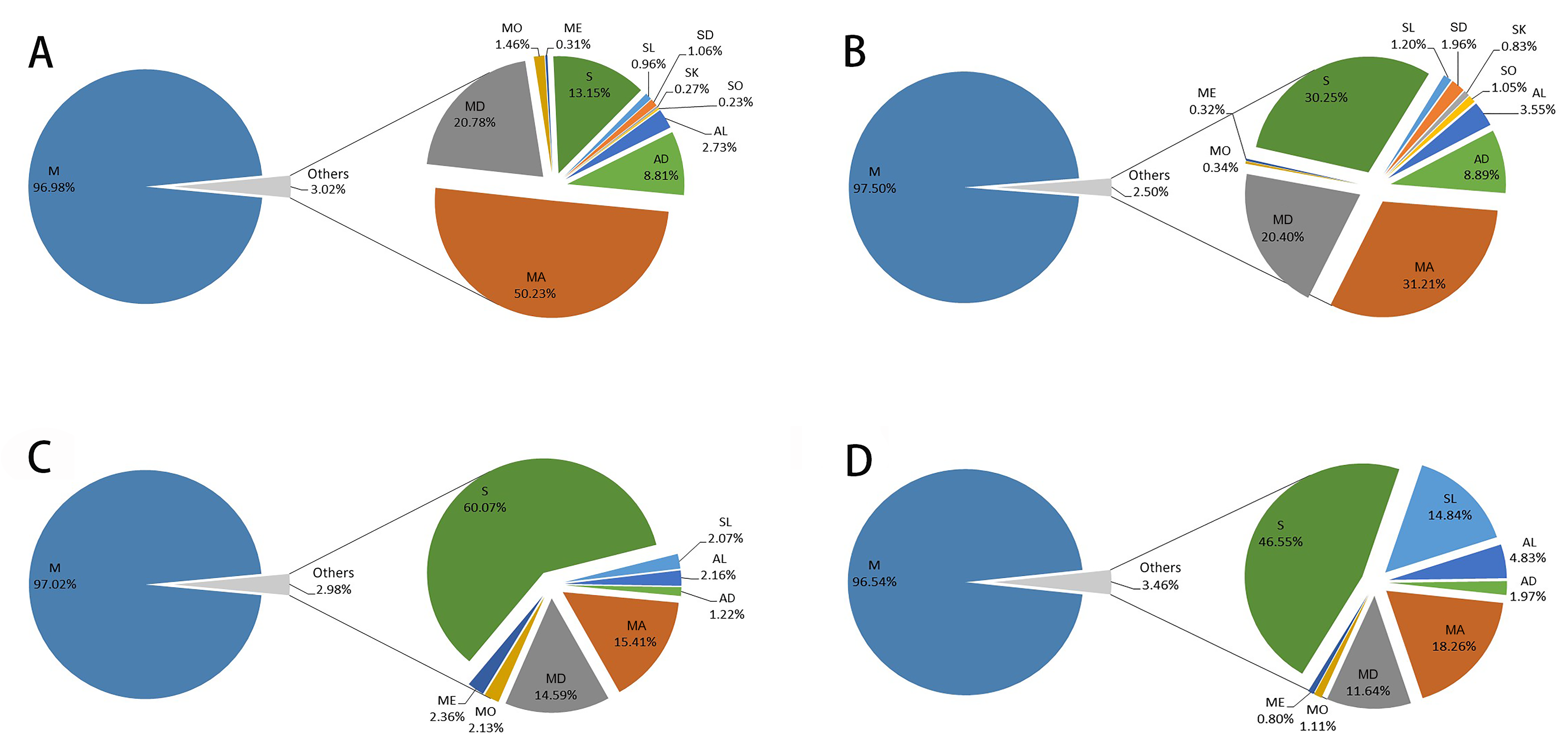

Supplement: Supplementary file 1 [file molecules-24-03456-s001.zip › molecules-585719-supplementary/Fig. S1.tif]
